# Weighted gene correlation network analysis identifies microenvironment-related genes signature as prognostic candidate for Grade II/III glioma

**DOI:** 10.18632/aging.104075

**Published:** 2020-11-07

**Authors:** Yong Li, Gang Deng, Huikai Zhang, Yangzhi Qi, Lun Gao, Yinqiu Tan, Ping Hu, Yixuan Wang, Baohui Liu, Qianxue Chen

**Affiliations:** 1 Department of Neurosurgery, Renmin Hospital of Wuhan University, Wuchang, Wuhan 430060, Hubei, P.R. China

**Keywords:** microenvironment, ESTIMATE algorithm, WGCNA, LASSO

## Abstract

Glioma is the most common malignant tumor in the central nervous system. Evidence shows that clinical efficacy of immunotherapy is closely related to the tumor microenvironment. This study aims to establish a microenvironment-related genes (MRGs) model to predict the prognosis of patients with Grade II/III gliomas. Gene expression profile and clinical data of 459 patients with Grade II/III gliomas were extracted from The Cancer Genome Atlas. Then according to the immune/stromal scores generated by the ESTIMATE algorithm, the patients were scored one by one. Weighted gene co-expression network analysis (WGCNA) was used to construct a gene co-expression network to identify potential biomarkers for predicting the prognosis of patients. When adjusting clinical features including age, histology, grading, IDH status, we found that these features were independently associated with survival. The predicted value of the prognostic model was then verified in 440 samples in CGGA part B dataset and 182 samples in CGGA part C dataset by univariate and multivariate cox analysis. The clinical samples of 10 patients further confirmed our signature. Our findings suggested the eight-MRGs signature identified in this study are valuable prognostic predictors for patients with Grade II/III glioma.

## INTRODUCTION

Gliomas are the most common malignant tumor type of the central nervous system which account for about 80% of primary malignant brain tumors [1]. According to the criteria of the World Health Organization (WHO), gliomas are divided into four grades (WHO Grade I, II, III and IV) [2]. WHO Grade I gliomas, mainly including pilocytic astrocytomas, are considered to be benign tumors [3]. WHO grade II/III gliomas tumors that include infiltrative astrocytomas and oligodendrogliomas mostly occur in the adult cerebral hemisphere [4]. The median overall survival (OS) of patients with Grade II glioma patients is about 6-12 years, while the patients with Grade III glioma is reduced to 3 years (30-40 months) [5, 6]. WHO grade IV glioblastoma multiforme (GBM), the most malignant glioma type with, accounts for approximately 50% of glioma cases, and has a median survival time of 14.2 months [7]. Although surgical treatment combined with radiotherapy and chemotherapy methods has made great strides, the overall prognosis of patients with glioma has not significantly been improved [8]. The social burden of Grade II/III gliomas is more serious due to the poor quality of life caused by surgery and the high recurrence rate. However, the current research is mostly focused on the GBM, but research on the Grade gliomas is very limited.

In recent years, researchers have made remarkable progress in the field of immunotherapy in blood and many other solid tumors, which provide a new choice for the glioma therapy [9–11]. Through immunotherapy, especially immune checkpoint inhibitors, significant responses were observed in different types of tumors [12, 13]. More and more evidence show that the effect of immunotherapy is not only associated with tumor cells, but also to the tumor microenvironment (TME). The tumor microenvironment refers to the surrounding tissue around tumor cells, including cytokines hormones, extracellular matrix and a series of immune and stromal cells, which have important influence on gene expression and clinical prognosis [14]. In view of these latest findings, novel therapies for immune responses are at present being developed and glioma microenvironment as a potential therapeutic target has been paid more and more attention [15–17].

With the development of sequencing technology, high-throughput genomic analysis platforms provide promising tools for clinical oncology research. Compared with traditional clinical characteristics, genomics indicators are uniform and objective. Multiple genes signature can be used in combination to provide additional prognostic information [18–21]. Yoshihara et al. calculate the expression of specific molecular biomarkers in immune cells and stromal cells to generate an estimation algorithm with immune/stroma/ESTIMATE score to predict the matrix components and immune cell infiltrating cells in TME [22]. The immune score is closely related to the degree of immune cell infiltration. The higher the scores, the more immune cells infiltration. The stromal score stands for tumor matrix components. The higher the score, the more the matrix around the tumor. The ESTIMATE score is the sum of the immune score and the stromal score. The higher the score, the lower the purity of the tumor. This method is the most widely used and most convincing method currently used, and it has been experimentally confirmed that it is consistent with the actual situation of the tissue surrounding the tumor [23, 24].

The weighted gene co-expression network analysis (WGCNA) is a powerful tool to construct free-scale gene co-expression networks which is widely used to analyze large-scale datasets [25]. Thus, we establish Grade II/III glioma prognosis signature, and investigated the clinical application of the model using the microenvironment-related genes (MRGs).

## RESULTS

### Immune scores and stromal scores are significantly associated with Grade II/III glioma patients’ overall survival

**Figure 1:**
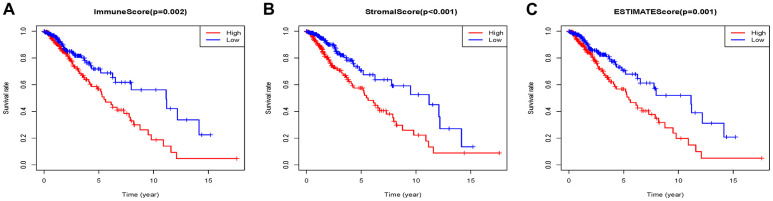
Associations of immune (**A**), stromal (**B**), and ESTIMATE (**C**) scores on overall survival in Grade II/III gliomas.

459 patients with Grade II/III glioma in the TCGA database with a follow-up time greater than 3 months were included in the present research. The stromal scores ranged from -1816.74 to 1716.82, immune scores were -1722.93 ~ 2189.02, and ESTIMATE scores were ~3512.4 to 3775.10 according to the ESTIMATE algorithm. K-M analysis was done to reveal the correlation between the scores and survival by dividing 459 patients into high and low score groups with the median score (low score group:230; high score group:229). The overall survival of Grade II/III glioma patients with low immune scores was significantly higher than that of patients with high immune scores (P=0.002) (Figure 1A). The same trend occurs in the high stromal (P < 0.001) (Figure 1B) and ESTIMATE (P=0.0012) (Figure 1C) score group.

### Co-expression module construction

**Figure 2:**
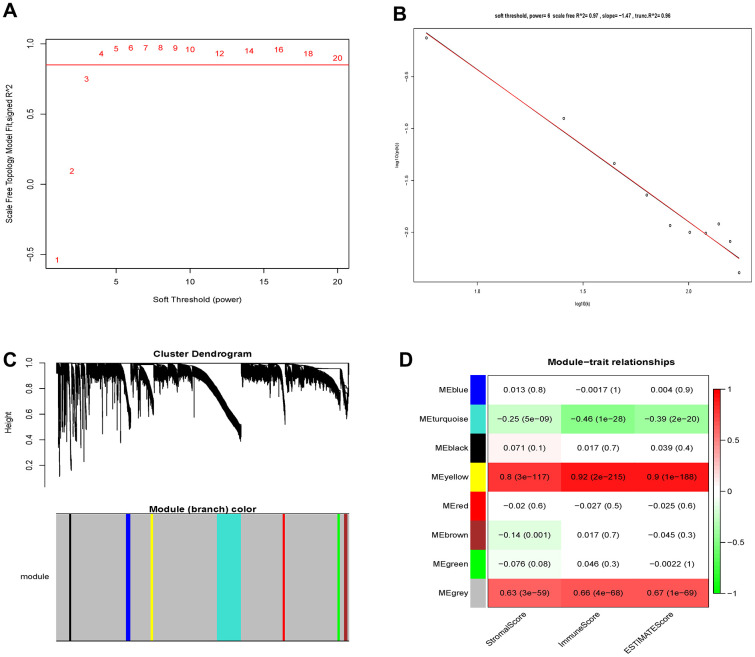
**Determination of soft-thresholding power and identification of modules associated with ESTIMATE scores in WGCNA.** (**A**) Analysis of the scale-free fit index for various soft-thresholding powers (β). (**B**) Checking the scale-free topology when β=6. (**C**) Dendrogram of all differentially expressed genes clustered based on a dissimilarity measure (1-TOM). (**D**) Heatmap of the correlation between module eigengenes and clinical traits of Grade II/III glioma cancer.

**Figure 3:**
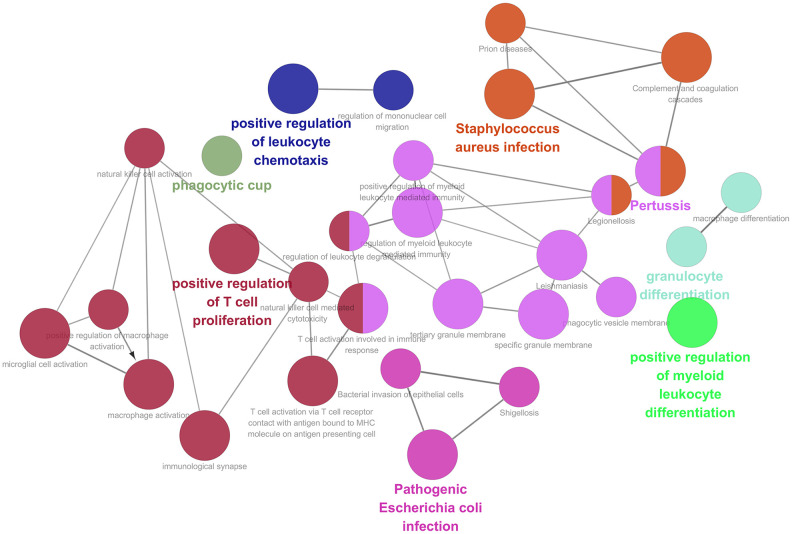
**Function enrichment analysis based on PPI network.** Functional annotations indicated that 46 genes in yellow modules were mostly involved in positive regulation of leukocyte chemotaxis, T cell proliferation and myeloid leukocyte differentiation.

459 samples from TCGA were clustered using the average linkage method to assess microarray quality and filter outliers. The power of β = 6 was chose as the soft-threshold (scale-free R2 = 0.97; Figure 2A–2C). 7 modules were clustered by average linkage hierarchical clustering. Yellow module was selected for further analysis, which was highly correlated with ESTIMATE score (Figure 2D). The enrichment analysis conducted in this study indicated that the genes in the yellow module were enriched in the positive regulation of leukocyte chemotaxis, myeloid leukocyte differentiation, and T cell proliferation (Figure 3). We did not select this grey module for further analysis for it contained too many genes to be processed in the next step, although the module was also significantly related to the ESTIMATE scores.

### Identification of a MRGs signature

**Figure 4:**
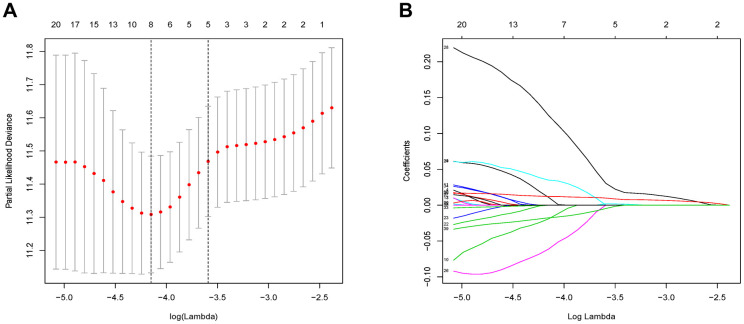
**Flow chart and 10-fold cross-validation for tuning parameter selection.** (**A**) 10-fold cross-validation for tuning parameter selection in the Lasso model. (**B**) LASSO coefficient profiles of the 46 prognostic genes. A vertical line is drawn at the value chosen by 10-fold cross-validation.

**Figure 5:**
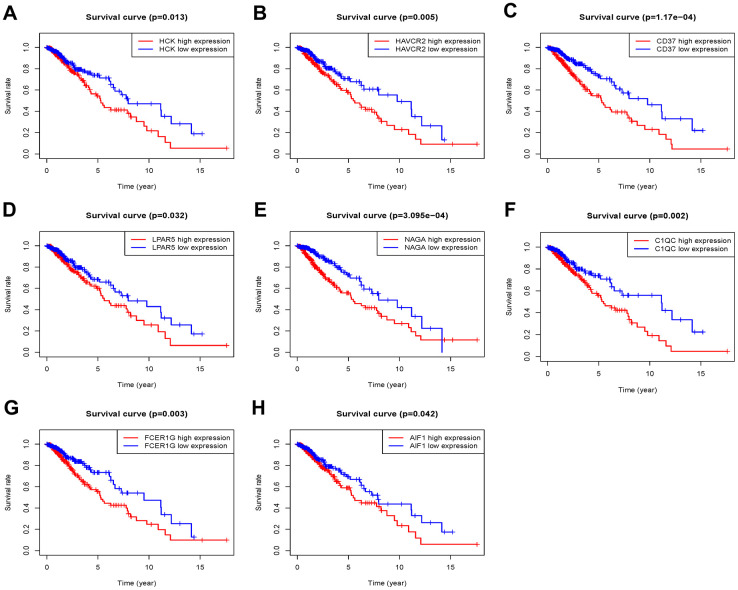
**Overall survival of genes in Grade II/III glioma patients in the TCGA cohort.** (**A**) HCK. (**B**) HAVCR2. (**C**) CD37. (**D**) LPAR5. (**E**) NAGA. (**F**) C1QC. (**G**) FCER1G. (**H**) AIF1.

**Figure 6:**
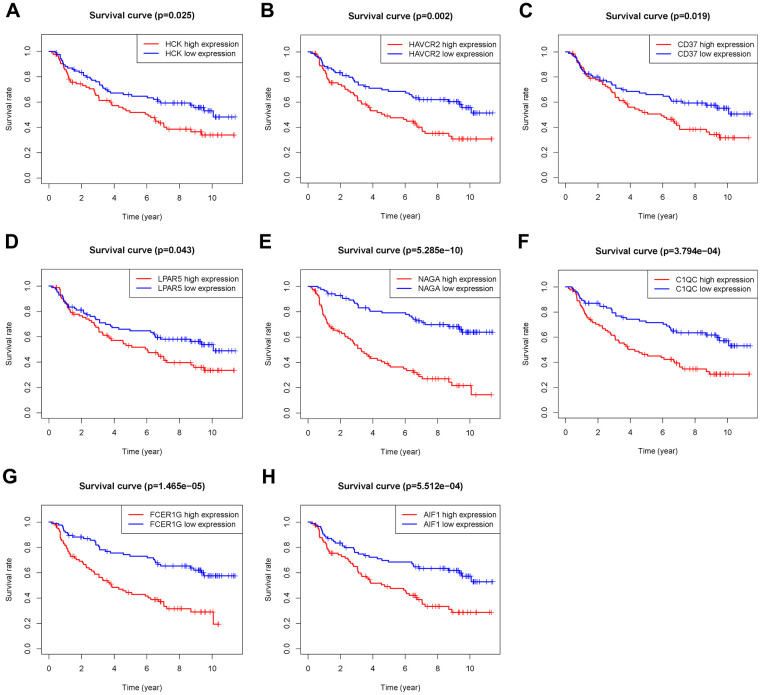
**Overall survival of genes in Grade II/III glioma patients in the CGGA Part C cohort.** (**A**) HCK. (**B**) HAVCR2. (**C**) CD37. (**D**) LPAR5. (**E**) NAGA. (**F**) C1QC. (**G**) FCER1G. (**H**) AIF1.

The 46 genes in the yellow module were considered to be related to the ESTIMATE scores and were conduct to the further research. Then Lasso-penalized Cox analysis with 10-fold cross-validation was performed to narrow the genes for prediction of the OS (Figure 4A). Eight microenvironment-related genes were identified (Figure 4B). Kaplan-Meier analysis in the TCGA and CGGA part C dataset indicated that all the 8 genes significant correlation with survival time of the patients (Figures 5, 6). The predictive model was defined as the linear combination of the expression levels of the 8 MRGs signature weighted by their relative coefficient in the multivariate Cox regression model, as risk score = (-0.092 * expression level of HCK) + (0.101 * expression level of HAVCR2) + (0.063 * expression level of CD37) + (−0.128 * expression level of LPAR5) + (0.290 * expression level of NAGA) + (−0.005 * expression level of C1QC) + (0.023 * expression level of FCER1G) + (−0.040 * expression level of AIF1). Risk score is determined by MRGs combined with survival time in TCGA dataset. The higher the risk score, the greater the ESTIMATE score, which means more immune cell infiltration and more stromal composition, and the shorter the survival time.

### Analysis of the MRGs signature in the TCGA cohort and CGGA cohort

**Figure 7:**
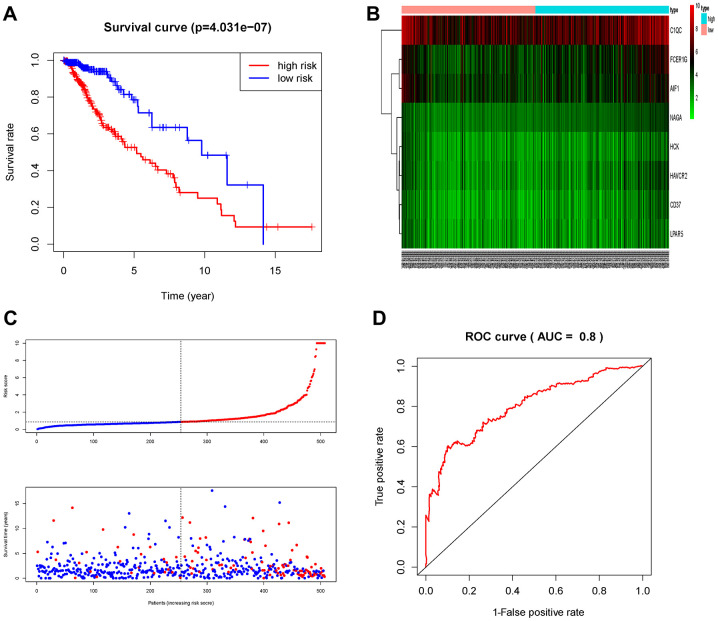
**Prognostic analysis of the TCGA cohort.** (**A**) Kaplan-Meier curve analysis of the high-risk and low-risk groups. (**B**) Expression patterns of risk genes in the prognostic model. (**C**) Risk score distribution of patients in the prognostic model and survival status scatter plots for patients in the prognostic model. (**D**) ROC curve analysis of the prognostic model.

The patients in the TCGA cohort were divided into high-risk group (n=229) and low-risk group (n=230) according to the risk score. We then evaluated the prognostic difference between the two groups by Kaplan-Meier curve based on the log-rank test. The results showed that the prognosis of the high-risk group was far worse than that of the low-risk group (P<0.001) (Figure 7A). We also made a heat map to describe the expression modules of MRGs in the two groups (Figure 7B). Then we sorted the risk scores of patients in the TCGA cohort, and marked the survival time and survival status of the samples in the dot plot. We found that the number of deaths in the high-risk group was much greater than that in the low-risk group, and their survival time was generally lower than that of the low-risk group. Then we performed ROC curve analysis to evaluate the ability of the MRGs signature as a detection method, and its area under the curve (AUC) reached 0.8 (Figure 7D), which illustrated the reliability of our MRGs signature.

After evaluating the expression characteristics of MRGs, we further performed univariate and multivariate Cox regression analysis to evaluate the role of MRGs signature in the prognosis of glioma patients. Univariate Cox analysis showed that MRGs risk score, age, the histology, the glioma grade, IDH gene mutant status and Karnofsky_score were closely associated to the prognosis of patients with Grade II/III glioma. The variables with P<0.05 in the univariate analysis were used for multivariate Cox analysis, and the results showed that MRGs signature were an independent risk factor when the clinical characteristics above were taken into consideration. (Table 1). In the CGGA part B and C cohorts, the MRGs risk score was still an independent prognostic factor (Tables 2, 3). These results indicated that the MRGs signature could be an independent prognosis characteristic of the patients with Grade II/III glioma.

**Table 1:** Univariate and multivariate analysis of prognosis factors in TCGA.

**Variables**	**Univariate**		**Multivariate**	
	**HR (95%CI)**	P*	**HR (95%CI)**	P*
Age(<45vs≥45)	3.252(2.157,4.902)	<0.001	3.519(2.227,5.559)	<0.001
Gender (Male vs Female)	0.958(0.653,1.405)	0.825		
Race (White vs Black)	1.650(0.764,3.560)	0.202		
Grade (G2 vs G3)	3.174(2.084,4.834)	<0.001	2.154(1.293,3.586)	0.003
Histology (Astrocytoma vs Oligodendroglioma)	0.532(0.343,0.824)	0.005	0.512(0.313,0.839)	0.008
IDH_mutant (Yes vs No)	6.866(2.138,22.05)	0.001	3.800(1.245,11.60)	0.019
K_SCORE (<90 vs≥90)	0.423(0.267,0.669)	<0.001	0.491(0.297,0.812)	0.006
Chemotherapy(Yes vs No)	0.549(0.304,0.991)	0.046	1.455(0.738,2.868)	0.279
Radiation (Yes vs No)	0.487(0.269,0.881)	0.017	1.498(0.733,3.062)	0.268
Risk_score (Low vs High)	3.252(2.046,5.168)	<0.001	2.713(1.675,4.394)	<0.001

HR: Hazard ratio; CI: Confidence interval.

**Table 2:** Cox proportional hazards regression model analysis of overall survival in CGGA Part B.

**Variable**	**Univariate analysis**			**Multivariate analysis**	
	**Wald**	**P**		**Wald**	**P**
Risk_score	19.623	<0.001		13.265	<0.001
Gender (Male vs Female)	0.213	0.645			
Age	0.734	0.391			
Grade (II vs III)	37.623	<0.001		38.283	<0.001
Histology	34.323	<0.001		0.308	0.579
IDH1_mutant (Yes vs No)	19.332	<0.001		0.020	0.889
1p19q_codeletion_status (Yes vs No)	27.873	<0.001		0.391	0.532
Radiotherapy (Yes vs No)	3.007	0.083		0.047	0.829
Chemotherapy (Yes vs No)	0.480	0.488			
MGMT	1.607	0.205			

**Table 3:** Univariate and multivariate analysis of prognosis factors in CGGA Part C dataset.

**Variables**	**Univariate**			**Multivariate**	
	**HR (95%CI)**	P*		**HR (95%CI)**	P*
Age(<45vs≥45)	1.877(1.210,2.912)	0.005		1.312(0.760,2.260)	0.330
Gender (Male vs Female)	1.510(0.982,2.322)	0.061			
Grade (G2 vs G3)	3.688(2.373,5.730)	<0.001		4.201(2.349,7.513)	0.003
Histology (Astrocytoma vs Oligodendroglioma)	0.151(0.067,0.343)	<0.001		0.184(0.075,0.451)	<0.001
IDH_mutant (Yes vs No)	2.691(1.720,4.210)	<0.001		1.146(0.633,2.075)	0.653
Chemotherapy (Yes vs No)	2.395(1.488,3.854)	<0.001		1.067(0.604,1.886)	0.824
Radiation (Yes vs No)	0.449(0.248,0.816)	0.009		0.383(0.201,0.730)	0.003
Risk_score (Low vs High)	3.133(1.984,4.947)	<0.001		1.958(1158,3.310)	0.012

**Figure 8:**
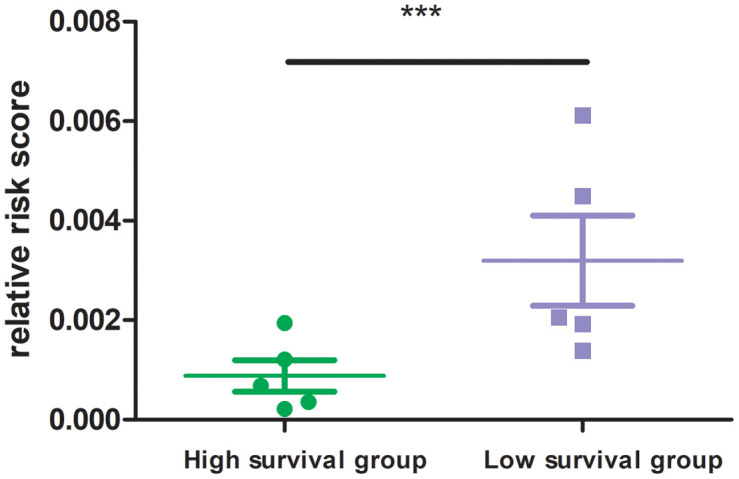
**Risk score of high and low survival time group.** 8 MRGs mRNA level was performed by quantitative PCR. GAPDH was used for normalization.

In experimental verification, we randomly selected 10 cases with a follow-up time of more than 3 months, and divided them into two groups according to their survival time (5 patients per group). All sample information is shown in the Supplementary Table 1. QPCR experiment was carried out to measure the mRNA expression of 8 MRGs in the ten patients. The results showed that the high survival group had lower risk score than that of the low survival group (Figure 8; P<0.001). This experiment verified the good clinical application value from another aspect of our MRGs signature.

### Immune infiltration of risk score

**Figure 9:**
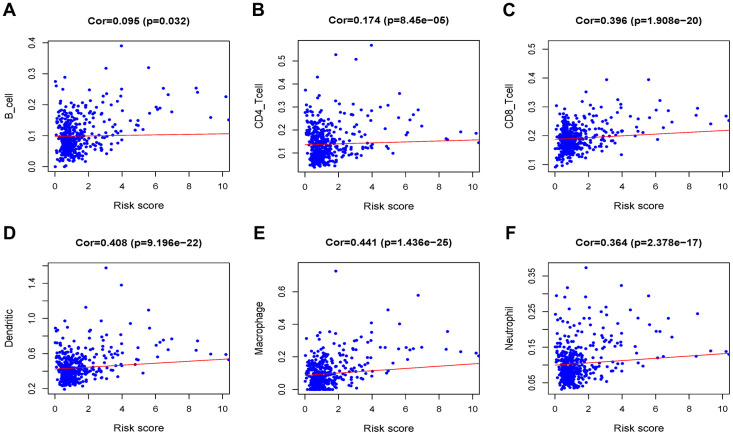
**Analysis of the correlation between the risk score and immune cell infiltration in the TCGA cohort.** (**A**) B cells. (**B**) CD4+ T cells. (**C**) CD8+ T cells. (**D**) Dendritic cells. (**E**) Macrophages. (**F**) Neutrophils.

After identifying prognostic value of the risk score, we performed correlation analysis between the MRGs signature and immune cell infiltration for Grade II/III glioma in Figure 9. The risk score was significantly correlated with the infiltration of CD4 + T cells, CD8 + T cells, dendritic cells, macrophages and neutrophil cells (all P < 0.05), suggesting that the level of immune infiltration was generally increased.

## DISCUSSION

Solid tumors consist of tumor cells and non-tumor tissue. Non-tumor tissue outside of tumor cells is often referred to as the tumor microenvironment. The composition of the solid tumor is usually determined by the pathologist through visual assessment, which may be affected by histopathological sensitivity, interobserver variability, and accuracy. With the development of high-throughput sequencing platform, the research on molecular mechanism of tumor is changing with each passing day. In addition to routine pathological assessments, advances in genomics have introduced many computational methods based on polygenic data to determine the tumor microenvironment [26]. These methods are highly consistent with pathologists' observations and are widely used in the study of various tumors.

Previous researches have studied the value of microenvironment-related gene signal models in predicting the prognosis of patients with different types of malignant tumors. Qi established a prognostic model based on 9 microenvironment-related genes and predicted the survival of patients with ovarian cancer. The results showed that the model could independently distinguish patients with different death risks. They further studied the relationship between these nine genes and immune cell infiltration, supporting the important role of the ovarian cancer TME in the occurrence and development of tumor cells. Xie et al. used 12 microenvironment-related genes to construct a breast cancer prognostic risk model and found that the model can accurately stratify patients with different survival outcomes [27]. All of these indicate that tumor microenvironment-related genes play key roles in the tumor prognosis, and are prognostic indicators potentially. However, the value of microenvironment-related genes for Grade II/III gliomas remains to be elucidated.

Through high-throughput expression profiling, WGCNA, lasso, and multivariate Cox analysis, we acquired eight genes (HCK, HAVCR2, CD37, LPAR5, NAGA, C1QC, FCER1G and AIF1) to construct the MRGs signature in Grade II/III glioma patients. We first evaluated characteristics of the microenvironment-related genes in TCGA and found that the survival rate in the high-risk group was significantly lower than that in the low-risk group which indicated that the MRGs signature could accurately stratify patients with different survival outcomes. The ROC analysis results showed the signature had a good predictive effect on glioma prognosis. In addition, the multivariate Cox regression model showed that the MRGs signature was an independent risk factor when the clinical characteristics such as age, pathological type, grade, IDH mutation, and K-score were taken into consideration. Similar results were observed when verifying in the CGGA Part B and Part C cohort. These results indicated that the MRGs signature was a novel and important pathway in predicting tumor immune infiltration and patient survival outcome in Grade II/III glioma. predict survival and. It may be utilized as a prognosis stratification tool added to the current system due to the good prospect of clinical application.

Previous studies have shown that immune infiltration is an important factor in determining glioma response and prognosis. We analyzed the correlation between the MRGs signature and infiltration of immune cells. Convincingly, we found that the MRGs risk score was positively correlated with several common types of infiltration immune cells (B cells, CD4 + T cells, CD8 + T cells, neutrophils, macrophages and dendritic cells). Further research needs to be done to explore the relationship among MRGs and immune infiltration and the impact of glioma prognosis.

The biological functions of some MRGs have been elucidated in glioma and other tumors. The protein expressed by HCK is a tyrosine kinase in Src family. This protein is mainly present in the hematopoietic system, especially in B-lymphoid and myeloid lineages cells. It may play a critical role in helping couple Fc receptors in immune cells [28]. HCK protein regulates the phagocytosis of neutrophils through the G protein-coupled IL8 receptor. In macrophages, HCK protein can regulate the immune response by enhancing their proliferation, migration and secretion of IL6 and the macrophages of the HCK knockout mice showed weak phagocytosis [29, 30]. Previous studies have shown that in leukemia breast and colon cancer the up-regulation of HCK protein is often accompanied by higher tumor grades and the prognosis of patients is mostly poor [31–33]. But the role of HCK in glioma is still unknown. The protein encoded by HAVCR2 (also named Tim-3) belongs to the immunoglobulin superfamily, and TIM family. It is a Th1-specific cell surface protein that regulates macrophage activation, and inhibits Th1-mediated auto- and alloimmune responses, and promotes immunological tolerance [34]. TTim-3 along with PD-1 marks exhausted CD4 and CD8 T cells in experimental models and in humans [35–37]. Studies indicated that the percentage of Tim-3 and PD-1 co-expressing T cells in tumors and blood of GBM patients increased compared with healthy group [38]. Although anti-Tim-3 alone is not enough to produce a therapeutic response, the combined application of anti-Tim-3 and anti-PD-1 antibodies can significantly improve the survival rate of a syngeneic mouse orthotopic glioma model [39]. CD37 is a member of the tetra-spanning superfamily and is involved in the cell adhesion, movement, differentiation, intercellular communication and immune response. In addition, it also participates the interaction between B and T cells and the production of immunoglobulin G [40]. CD37 is highly expressed on the surface of most CLL and NHL cases, which is an attractive target for immunotherapy [41]. However, its role in glioma is still unclear. LPAR5 acts as a rhodopsin class of G protein-coupled transmembrane receptors [42]. ATX-LPAR signaling axis is reported to induce MMP-9 expression in hepatocellular carcinoma (HCC) subsequently enhancing the invasive capacity of these cells [43]. NAGA encodes the lysosomal enzyme alpha-N-acetylgalactosaminidase, which cleaves alpha-N-acetylgalactosaminyl moieties from glycoconjugates [44]. At present, there are very few researches on NAGA. C1QC protein is the C-chain polypeptide of C1q. C1QC is mainly involved in complement-mediated innate and adaptive immune response. Study showed that C1q promotes the maturation of dendritic cells (DC) through the combination of pathogen-associated molecular patterns and danger-related molecular patterns, and then interacts with different molecules on the surface of dendritic cells in the early stages of immunity [45]. FCER1G, also known as FcRγ, is a component of the immunoglobulin E receptor and IL-3 receptor complex. FCER1G mainly participates in biological processes of allergic inflammation signals and can regulate the activation of neutrophils and platelets [46]. FCER1G plays a tumor-promoting role in various tumors such as leukemia and solid tumors of the renal and Meninges [47–50]. AIF1, also called IBA1, encodes a protein that binds actin and calcium. The function of AIF1 protein is closely related to the activation of macrophages and is a specific marker for the activation of macrophages and microglia in the central nervous system [51]. It is reported that in hepatocellular carcinoma macrophages overexpressing AIF1 promoted tumor migration and proliferation of tumor cells [52]. In vitro study showed that AIF1 promoted tumor growth via the NF-κB/cyclin D1 pathway [53]. The biological roles of most MRGs remain largely unclear in Grade II/III glioma. Further studies are required to explore the underlying mechanisms of the MRGs on glioma.

There are several differences between our work and previous research. First, we focused on the microenvironment-related genes expression pattern in Grade II/III gliomas using ESTIMATE algorithm. This is the first application of the algorithm to Grade II/III gliomas. Secondly, unlike articles with other single analysis methods, we use multiple algorithms (including estimation algorithms, WGCNA, and lasso regression) to identify microenvironment-related genes. Finally, the results were verified using two independent databases and a small sample experiment, making our conclusion more reliable.

Inevitably, our research has some shortcomings. Firstly, we use the retrospective data from public databases that have not been validated in prospective study. Secondly, the mechanisms of the MRGs on Grade II/III glioma needs further experimental research in vitro and in vivo. Additionally, due to the small number of patients in experimental verification, the signature still needs to be verified by larger samples.

## CONCLUSIONS

To sum up, we discovered 8 microenvironment related genes, and constructed a signature to group patients at low and high risk of low survival time. This feature may have potential significance in evaluating the prognosis of Grade II/III glioma patients.

## MATERIALS AND METHODS

### Data processing

The TCGA -LGG level 3 gene expression profiles and the clinical data of 529 samples of Grade II/III glioma patients were acquired from the TCGA data coordination center (https://portal.gdc.cancer.gov/) [54]. A total of 19767 protein coding genes were quantified by fragments per kilobase of exon per million reads mapped (FPKM). The ESTIMATE algorithm in the R project was applied to calculate the stromal and immune scores of each glioma sample in the TCGA dataset. 440 Grade II/III glioma samples including RNA sequencing in the CGGA part B dataset and 182 Grade II/III glioma samples in the CGGA part C dataset was acquired to verify the signature (http://www.cgga.org.cn/) [55, 56]. Cases with a follow-up time greater than 3 months were used for later analysis. 10 simples from Renmin Hospital of Wuhan University with clinical data was used for experimental validation.

### Co-expression network construction and identification of clinical significant modules

The R package ‘limma’ was applied to preprocess the downloaded raw data including background adjustment and normalization. Using the R package ‘WGCNA’, we further processed the dataset by variance analysis. 14828 genes by P-value were screened out due to the low sensitive of the WGCNA analysis in low expression changes genes amid samples. The remaining 4895 genes were chosen for further analysis. The gene co-expression network was constructed by WGCNA package in R using the expression data profile of these 4 895 genes. 6 was selected as the soft threshold to convert the co-expression similarity matrix into an adjacency matrix. Then the topological matrix was developed by using the topological overlap measure (TOM) in the R project. The minimum size of genes for each module was set as 30. The feature genes of modules are calculated, and the similar modules are clustered and merged according to the module dissection threshold. Finally, the correlations between gene modules and clinical traits were calculated and visualized through a heatmap. The modules which is positively related to ESTIMATE scores was selected for further analysis.

### Gene ontology and pathway enrichment analysis

ClueGO (v2.5.5) is a Cytoscape (v3.7.1) plugin that visualizes the non-redundant biological terms for large clusters of genes in a functionally grouped network. CluePedia (v1.5.3) is a functional module of ClueGO [57, 58]. Biological process (BP), cellular component (CC) and molecular function (MF) functional groups in GO terms and KEGG pathways analysis of selected genes were enrolled and visualized using ClueGO and CluePedia. The pathways with adj-P < 0.05 were visualized in Cytoscape.

### Construction of the MRGs prognostic signature

The Least Absolute Shrinkage Sum Selection Operator (LASSO) is an analysis method that accurately sets the regression coefficients of many irrelevant features to zero, thereby reducing interference variables. It is an important method and has many applications in regression analysis [59, 60]. The LASSO regression analysis was performed by R package ‘glmnet’. In the analysis, genes in the module which is positively related to ESTIMATE scores were used as the input. Eight genes were got from the LASSO regression. Then, multivariate Cox regression analysis was performed to evaluate the weight coefficient of the genes and constructed a prognostic risk model.

$$\begin{array}{l} \text{Risk}\;\text{score}\;(\text{patient})=\\ \;\sum_{i=1}^{n}coefficient\;(gene\;i)\;expression\;value\;of\;(gene\;i) \end{array}$$

### ESTIMATE scores-based gene signature

Overall survival was analyzed by R package ‘survival’ using Kaplan-Meier method, and the log-rank test was performed to explore qualitative variables as appropriate of the differences of survival between groups. Time-dependent receiver operating characteristics (ROC) analysis using the R package ‘survivalROC’ was carried out to investigate the prognostic accuracy of MRGs signature. Immune infiltrate data of Grade II/III glioma cases containing the level of 6 types of tumor-infiltrating immune cells (B cells, CD4+ T cells, CD8+ T cells, neutrophils, macrophages and dendritic cells) was obtained from the Tumor Immune Estimation Resource (https://cistrome.shinyapps.io/timer/) [61].

### Quantitative polymerase chain reaction

10 human glioma simples were obtained from the Department of Neurosurgery, Renmin Hospital of Wuhan University. Quantitative PCR of the 8 genes mRNA level was perform using Bio Rad CFX Connect Real-Time System. GAPDH was used for normalization.

### Statistical analyses

Statistical analyses were performed using SPSS v25.0 (SPSS Inc. Chicago, IL, U.S.A.). The univariate and multivariate analysis was performed by cox regression model. Hazard ratios (HRs) with their respective 95% confidence intervals were calculated by Wald test. All tests were two-sided and P <0.05 was considered statistically significant.

### Ethical approval

This study was approved by the Institutional Ethics Committee of the Faculty of Medicine at Renmin Hospital of Wuhan University [approval number: 2012LKSZ (010) H].

## Supplementary Material

Supplementary Table 1

## References

[1] Song X, Zhang N, Han P, Moon BS, Lai RK, Wang K, Lu W. Circular RNA profile in gliomas revealed by identification tool UROBORUS. Nucleic Acids Res. 2016; 44:e87. 10.1093/nar/gkw075 26873924 PMC4872085

[2] Louis DN, Perry A, Reifenberger G, von Deimling A, Figarella-Branger D, Cavenee WK, Ohgaki H, Wiestler OD, Kleihues P, Ellison DW. The 2016 world health organization classification of tumors of the central nervous system: a summary. Acta Neuropathol. 2016; 131:803–20. 10.1007/s00401-016-1545-1 27157931

[3] Bornhorst M, Frappaz D, Packer RJ. Pilocytic astrocytomas. Handb Clin Neurol. 2016; 134:329–44. 10.1016/B978-0-12-802997-8.00020-7 26948364

[4] Wesseling P, Capper D. WHO 2016 classification of gliomas. Neuropathol Appl Neurobiol. 2018; 44:139–50. 10.1111/nan.12432 28815663

[5] Rasmussen BK, Hansen S, Laursen RJ, Kosteljanetz M, Schultz H, Nørgård BM, Guldberg R, Gradel KO. Epidemiology of glioma: clinical characteristics, symptoms, and predictors of glioma patients grade I-IV in the the danish neuro-oncology registry. J Neurooncol. 2017; 135:571–79. 10.1007/s11060-017-2607-5 28861666

[6] Thon N, Kreth FW, Tonn JC. The role of surgery in grade II/III oligodendroglial tumors. CNS Oncol. 2015; 4:317–23. 10.2217/cns.15.26 26478133 PMC6082329

[7] Stupp R, Mason WP, van den Bent MJ, Weller M, Fisher B, Taphoorn MJ, Belanger K, Brandes AA, Marosi C, Bogdahn U, Curschmann J, Janzer RC, Ludwin SK, et al, and European Organisation for Research and Treatment of Cancer Brain Tumor and Radiotherapy Groups, and National Cancer Institute of Canada Clinical Trials Group. Radiotherapy plus concomitant and adjuvant temozolomide for glioblastoma. N Engl J Med. 2005; 352:987–96. 10.1056/NEJMoa043330 15758009

[8] Yang P, Wang Y, Peng X, You G, Zhang W, Yan W, Bao Z, Wang Y, Qiu X, Jiang T. Management and survival rates in patients with glioma in China (2004-2010): a retrospective study from a single-institution. J Neurooncol. 2013; 113:259–66. 10.1007/s11060-013-1103-9 23483435

[9] Blum S, Martins F, Lübbert M. Immunotherapy in adult acute leukemia. Leuk Res. 2017; 60:63–73. 10.1016/j.leukres.2017.06.011 28756350

[10] Steven A, Fisher SA, Robinson BW. Immunotherapy for lung cancer. Respirology. 2016; 21:821–33. 10.1111/resp.12789 27101251

[11] Sugie T. Immunotherapy for metastatic breast cancer. Chin Clin Oncol. 2018; 7:28. 10.21037/cco.2018.05.05 30056730

[12] Sui H, Ma N, Wang Y, Li H, Liu X, Su Y, Yang J. anti-PD-1/PD-L1 therapy for non-small-cell lung cancer: toward personalized medicine and combination strategies. J Immunol Res. 2018; 2018:6984948. 10.1155/2018/6984948 30159341 PMC6109480

[13] Waidmann O. Recent developments with immunotherapy for hepatocellular carcinoma. Expert Opin Biol Ther. 2018; 18:905–10. 10.1080/14712598.2018.1499722 29995439

[14] Wu T, Dai Y. Tumor microenvironment and therapeutic response. Cancer Lett. 2017; 387:61–68. 10.1016/j.canlet.2016.01.043 26845449

[15] Pitt JM, Marabelle A, Eggermont A, Soria JC, Kroemer G, Zitvogel L. Targeting the tumor microenvironment: removing obstruction to anticancer immune responses and immunotherapy. Ann Oncol. 2016; 27:1482–92. 10.1093/annonc/mdw168 27069014

[16] Frankel T, Lanfranca MP, Zou W. The role of tumor microenvironment in cancer immunotherapy. Adv Exp Med Biol. 2017; 1036:51–64. 10.1007/978-3-319-67577-0_4 29275464

[17] Gieryng A, Pszczolkowska D, Walentynowicz KA, Rajan WD, Kaminska B. Immune microenvironment of gliomas. Lab Invest. 2017; 97:498–518. 10.1038/labinvest.2017.19 28287634

[18] Long J, Zhang L, Wan X, Lin J, Bai Y, Xu W, Xiong J, Zhao H. A four-gene-based prognostic model predicts overall survival in patients with hepatocellular carcinoma. J Cell Mol Med. 2018; 22:5928–38. 10.1111/jcmm.13863 30247807 PMC6237588

[19] Song J, Xu Q, Zhang H, Yin X, Zhu C, Zhao K, Zhu J. Five key lncRNAs considered as prognostic targets for predicting pancreatic ductal adenocarcinoma. J Cell Biochem. 2018; 119:4559–69. 10.1002/jcb.26598 29239017 PMC5947154

[20] Wu M, Li X, Zhang T, Liu Z, Zhao Y. Identification of a nine-gene signature and establishment of a prognostic nomogram predicting overall survival of pancreatic cancer. Front Oncol. 2019; 9:996. 10.3389/fonc.2019.00996 31612115 PMC6776930

[21] Zuo Y, Zhang L, Tang W, Tang W. Identification of prognosis-related alternative splicing events in kidney renal clear cell carcinoma. J Cell Mol Med. 2019; 23:7762–72. 10.1111/jcmm.14651 31489763 PMC6815842

[22] Yoshihara K, Shahmoradgoli M, Martínez E, Vegesna R, Kim H, Torres-Garcia W, Treviño V, Shen H, Laird PW, Levine DA, Carter SL, Getz G, Stemke-Hale K, et al. Inferring tumour purity and stromal and immune cell admixture from expression data. Nat Commun. 2013; 4:2612. 10.1038/ncomms3612 24113773 PMC3826632

[23] Li J, Li X, Zhang C, Zhang C, Wang H. A signature of tumor immune microenvironment genes associated with the prognosis of non-small cell lung cancer. Oncol Rep. 2020; 43:795–806. 10.3892/or.2020.7464 32020214

[24] Meng Z, Ren D, Zhang K, Zhao J, Jin X, Wu H. Using ESTIMATE algorithm to establish an 8-mRNA signature prognosis prediction system and identify immunocyte infiltration-related genes in pancreatic adenocarcinoma. Aging (Albany NY). 2020; 12:5048–70. 10.18632/aging.102931 32181755 PMC7138590

[25] Langfelder P, Horvath S. WGCNA: an R package for weighted correlation network analysis. BMC Bioinformatics. 2008; 9:559. 10.1186/1471-2105-9-559 19114008 PMC2631488

[26] Zhang C, Cheng W, Ren X, Wang Z, Liu X, Li G, Han S, Jiang T, Wu A. Tumor purity as an underlying key factor in glioma. Clin Cancer Res. 2017; 23:6279–91. 10.1158/1078-0432.CCR-16-2598 28754819

[27] Xie P, Ma Y, Yu S, An R, He J, Zhang H. Development of an immune-related prognostic signature in breast cancer. Front Genet. 2020; 10:1390. 10.3389/fgene.2019.01390 32047513 PMC6997532

[28] Guiet R, Poincloux R, Castandet J, Marois L, Labrousse A, Le Cabec V, Maridonneau-Parini I. Hematopoietic cell kinase (Hck) isoforms and phagocyte duties - from signaling and actin reorganization to migration and phagocytosis. Eur J Cell Biol. 2008; 87:527–42. 10.1016/j.ejcb.2008.03.008 18538446

[29] Poh AR, O’Donoghue RJ, Ernst M. Hematopoietic cell kinase (HCK) as a therapeutic target in immune and cancer cells. Oncotarget. 2015; 6:15752–71. 10.18632/oncotarget.4199 26087188 PMC4599235

[30] Lowell CA, Berton G. Resistance to endotoxic shock and reduced neutrophil migration in mice deficient for the src-family kinases hck and fgr. Proc Natl Acad Sci USA. 1998; 95:7580–84. 10.1073/pnas.95.13.7580 9636192 PMC22689

[31] Clark SS, McLaughlin J, Timmons M, Pendergast AM, Ben-Neriah Y, Dow LW, Crist W, Rovera G, Smith SD, Witte ON. Expression of a distinctive BCR-ABL oncogene in Ph1-positive acute lymphocytic leukemia (ALL). Science. 1988; 239:775–77. 10.1126/science.3422516 3422516

[32] Rody A, Holtrich U, Pusztai L, Liedtke C, Gaetje R, Ruckhaeberle E, Solbach C, Hanker L, Ahr A, Metzler D, Engels K, Karn T, Kaufmann M. T-cell metagene predicts a favorable prognosis in estrogen receptor-negative and HER2-positive breast cancers. Breast Cancer Res. 2009; 11:R15. 10.1186/bcr2234 19272155 PMC2688939

[33] Leroy C, Fialin C, Sirvent A, Simon V, Urbach S, Poncet J, Robert B, Jouin P, Roche S. Quantitative phosphoproteomics reveals a cluster of tyrosine kinases that mediates SRC invasive activity in advanced colon carcinoma cells. Cancer Res. 2009; 69:2279–86. 10.1158/0008-5472.CAN-08-2354 19276381

[34] Das M, Zhu C, Kuchroo VK. Tim-3 and its role in regulating anti-tumor immunity. Immunol Rev. 2017; 276:97–111. 10.1111/imr.12520 28258697 PMC5512889

[35] Zhou E, Huang Q, Wang J, Fang C, Yang L, Zhu M, Chen J, Chen L, Dong M. Up-regulation of tim-3 is associated with poor prognosis of patients with colon cancer. Int J Clin Exp Pathol. 2015; 8:8018–27. 26339368 PMC4555696

[36] Yang M, Yu Q, Liu J, Fu W, Cao Y, Yu L, Shao S, Wang X, Niu H, Wang Y. T-cell immunoglobulin mucin-3 expression in bladder urothelial carcinoma: clinicopathologic correlations and association with survival. J Surg Oncol. 2015; 112:430–35. 10.1002/jso.24012 26265374

[37] Gonçalves Silva I, Gibbs BF, Bardelli M, Varani L, Sumbayev VV. Differential expression and biochemical activity of the immune receptor tim-3 in healthy and Malignant human myeloid cells. Oncotarget. 2015; 6:33823–33. 10.18632/oncotarget.5257 26413815 PMC4741805

[38] Goods BA, Hernandez AL, Lowther DE, Lucca LE, Lerner BA, Gunel M, Raddassi K, Coric V, Hafler DA, Love JC. Functional differences between PD-1+ and PD-1- CD4+ effector T cells in healthy donors and patients with glioblastoma multiforme. PLoS One. 2017; 12:e0181538. 10.1371/journal.pone.0181538 28880903 PMC5589094

[39] Kim JE, Patel MA, Mangraviti A, Kim ES, Theodros D, Velarde E, Liu A, Sankey EW, Tam A, Xu H, Mathios D, Jackson CM, Harris-Bookman S, et al. Combination therapy with anti-PD-1, anti-TIM-3, and focal radiation results in regression of murine gliomas. Clin Cancer Res. 2017; 23:124–36. 10.1158/1078-0432.CCR-15-1535 27358487 PMC5735836

[40] Payandeh Z, Noori E, Khalesi B, Mard-Soltani M, Abdolalizadeh J, Khalili S. anti-CD37 targeted immunotherapy of b-cell Malignancies. Biotechnol Lett. 2018; 40:1459–66. 10.1007/s10529-018-2612-6 30293139

[41] Deckert J, Park PU, Chicklas S, Yi Y, Li M, Lai KC, Mayo MF, Carrigan CN, Erickson HK, Pinkas J, Lutz RJ, Chittenden T, Lambert JM. A novel anti-CD37 antibody-drug conjugate with multiple anti-tumor mechanisms for the treatment of b-cell Malignancies. Blood. 2013; 122:3500–10. 10.1182/blood-2013-05-505685 24002446

[42] Lee SC, Fujiwara Y, Liu J, Yue J, Shimizu Y, Norman DD, Wang Y, Tsukahara R, Szabo E, Patil R, Banerjee S, Miller DD, Balazs L, et al. Autotaxin and LPA1 and LPA5 receptors exert disparate functions in tumor cells versus the host tissue microenvironment in melanoma invasion and metastasis. Mol Cancer Res. 2015; 13:174–85. 10.1158/1541-7786.MCR-14-0263 25158955 PMC4297753

[43] Park SY, Jeong KJ, Panupinthu N, Yu S, Lee J, Han JW, Kim JM, Lee JS, Kang J, Park CG, Mills GB, Lee HY. Lysophosphatidic acid augments human hepatocellular carcinoma cell invasion through LPA1 receptor and MMP-9 expression. Oncogene. 2011; 30:1351–59. 10.1038/onc.2010.517 21102517

[44] Salvayre R, Negre A, Maret A, Douste-Blazy L. [Alpha-galactosidases and alpha-N-acetylgalactosaminidase. Biochemical bases of fabry’s disease]. Pathol Biol (Paris). 1984; 32:269–84. 6326022

[45] Hosszu KK, Santiago-Schwarz F, Peerschke EI, Ghebrehiwet B. Evidence that a C1q/C1qR system regulates monocyte-derived dendritic cell differentiation at the interface of innate and acquired immunity. Innate Immun. 2010; 16:115–27. 10.1177/1753425909339815 19710097 PMC2846191

[46] Fu L, Cheng Z, Dong F, Quan L, Cui L, Liu Y, Zeng T, Huang W, Chen J, Pang Y, Ye X, Wu G, Qian T, et al. Enhanced expression of FCER1G predicts positive prognosis in multiple myeloma. J Cancer. 2020; 11:1182–94. 10.7150/jca.37313 31956364 PMC6959079

[47] Rajaraman P, Brenner AV, Neta G, Pfeiffer R, Wang SS, Yeager M, Thomas G, Fine HA, Linet MS, Rothman N, Chanock SJ, Inskip PD. Risk of meningioma and common variation in genes related to innate immunity. Cancer Epidemiol Biomarkers Prev. 2010; 19:1356–61. 10.1158/1055-9965.EPI-09-1151 20406964 PMC3169167

[48] Han S, Lan Q, Park AK, Lee KM, Park SK, Ahn HS, Shin HY, Kang HJ, Koo HH, Seo JJ, Choi JE, Ahn YO, Chanock SJ, et al. Polymorphisms in innate immunity genes and risk of childhood leukemia. Hum Immunol. 2010; 71:727–30. 10.1016/j.humimm.2010.04.004 20438785 PMC2967770

[49] Chen L, Yuan L, Wang Y, Wang G, Zhu Y, Cao R, Qian G, Xie C, Liu X, Xiao Y, Wang X. Co-expression network analysis identified FCER1G in association with progression and prognosis in human clear cell renal cell carcinoma. Int J Biol Sci. 2017; 13:1361–72. 10.7150/ijbs.21657 29209141 PMC5715520

[50] Handschuh L, Kaźmierczak M, Milewski MC, Góralski M, Łuczak M, Wojtaszewska M, Uszczyńska-Ratajczak B, Lewandowski K, Komarnicki M, Figlerowicz M. Gene expression profiling of acute myeloid leukemia samples from adult patients with AML-M1 and -M2 through boutique microarrays, real-time PCR and droplet digital PCR. Int J Oncol. 2018; 52:656–78. 10.3892/ijo.2017.4233 29286103 PMC5807040

[51] Nam SJ, Kim YH, Park JE, Ra YS, Khang SK, Cho YH, Kim JH, Sung CO. Tumor-infiltrating immune cell subpopulations and programmed death ligand 1 (PD-L1) expression associated with clinicopathological and prognostic parameters in ependymoma. Cancer Immunol Immunother. 2019; 68:305–318. 10.1007/s00262-018-2278-x 30483834 PMC11028367

[52] Cai H, Zhu XD, Ao JY, Ye BG, Zhang YY, Chai ZT, Wang CH, Shi WK, Cao MQ, Li XL, Sun HC. Colony-stimulating factor-1-induced AIF1 expression in tumor-associated macrophages enhances the progression of hepatocellular carcinoma. Oncoimmunology. 2017; 6:e1333213. 10.1080/2162402X.2017.1333213 28932635 PMC5599077

[53] Liu S, Tan WY, Chen QR, Chen XP, Fu K, Zhao YY, Chen ZW. daintain/AIF-1 promotes breast cancer proliferation via activation of the NF-kappaB/cyclin D1 pathway and facilitates tumor growth. Cancer Sci. 2008; 99:952–57. 10.1111/j.1349-7006.2008.00787.x 18341653 PMC11159275

[54] Ceccarelli M, Barthel FP, Malta TM, Sabedot TS, Salama SR, Murray BA, Morozova O, Newton Y, Radenbaugh A, Pagnotta SM, Anjum S, Wang J, Manyam G, et al, and TCGA Research Network. Molecular profiling reveals biologically discrete subsets and pathways of progression in diffuse glioma. Cell. 2016; 164:550–63. 10.1016/j.cell.2015.12.028 26824661 PMC4754110

[55] Bao ZS, Chen HM, Yang MY, Zhang CB, Yu K, Ye WL, Hu BQ, Yan W, Zhang W, Akers J, Ramakrishnan V, Li J, Carter B, et al. RNA-seq of 272 gliomas revealed a novel, recurrent PTPRZ1-MET fusion transcript in secondary glioblastomas. Genome Res. 2014; 24:1765–73. 10.1101/gr.165126.113 25135958 PMC4216918

[56] Zhao Z, Meng F, Wang W, Wang Z, Zhang C, Jiang T. Comprehensive RNA-seq transcriptomic profiling in the malignant progression of gliomas. Sci Data. 2017; 4:170024. 10.1038/sdata.2017.24 28291232 PMC5349247

[57] Bindea G, Mlecnik B, Hackl H, Charoentong P, Tosolini M, Kirilovsky A, Fridman WH, Pagès F, Trajanoski Z, Galon J. ClueGO: a cytoscape plug-in to decipher functionally grouped gene ontology and pathway annotation networks. Bioinformatics. 2009; 25:1091–93. 10.1093/bioinformatics/btp101 19237447 PMC2666812

[58] Bindea G, Galon J, Mlecnik B. CluePedia cytoscape plugin: pathway insights using integrated experimental and in silico data. Bioinformatics. 2013; 29:661–63. 10.1093/bioinformatics/btt019 23325622 PMC3582273

[59] Tang J, Cui Q, Zhang D, Liao X, Zhu J, Wu G. An estrogen receptor (ER)- related signature in predicting prognosis of ER-positive breast cancer following endocrine treatment. J Cell Mol Med. 2019; 23:4980–90. 10.1111/jcmm.14338 31124293 PMC6652714

[60] Liang R, Zhi Y, Zheng G, Zhang B, Zhu H, Wang M. Analysis of long non-coding RNAs in glioblastoma for prognosis prediction using weighted gene co-expression network analysis, Cox regression, and L1-LASSO penalization. Onco Targets Ther. 2018; 12:157–168. 10.2147/OTT.S171957 30613154 PMC6306053

[61] Bhattacharya S, Andorf S, Gomes L, Dunn P, Schaefer H, Pontius J, Berger P, Desborough V, Smith T, Campbell J, Thomson E, Monteiro R, Guimaraes P, et al. ImmPort: disseminating data to the public for the future of immunology. Immunol Res. 2014; 58:234–39. 10.1007/s12026-014-8516-1 24791905

